# Nursing Interventions to Prevent Delirium in Critically Ill Patients in the Intensive Care Unit during the COVID19 Pandemic—Narrative Overview

**DOI:** 10.3390/healthcare8040578

**Published:** 2020-12-21

**Authors:** Dorota Ozga, Sabina Krupa, Paweł Witt, Wioletta Mędrzycka-Dąbrowska

**Affiliations:** 1 Institute of Health Sciences, Medical College of Rzeszow University, 35-310 Rzeszow, Poland; gdozga@poczta.fm (D.O.); sabinakrupa@o2.pl (S.K.); 2 Department of Pediatric Anesthesiology and Intensive Care, Medical University of Warsaw, 02-091 Warszawa, Poland; pawwitt@gmail.com; 3 Department of Anaesthesiology Nursing & Intensive Care, Medical University in Gdansk, 80-211 Gdańsk, Poland

**Keywords:** delirium, critical care, nurses, patients, SARS-CoV-2, intensive care units, personalized medicine

## Abstract

It has become a standard measure in recent years to utilise evidence-based practice, which is associated with a greater need to implement and use advanced, reliable methods of summarising the achievements of various scientific disciplines, including such highly specialised approaches as personalised medicine. The aim of this paper was to discuss the current state of knowledge related to improvements in “nursing” involving management of delirium in intensive care units during the SARS-CoV-2 pandemic. This narrative review summarises the current knowledge concerning the challenges associated with assessment of delirium in patients with COVID-19 by ICU nurses, and the role and tasks in the personalised approach to patients with COVID-19.

## 1. Introduction

The emergence of personalised, stratified, or precision medicine (PM) has introduced a new, previously unknown approach in healthcare aiming to identify and anticipate the optimal treatment outcome in a given patient. The older term, evidence-based practice (EBP) focuses on broad categories such as population averages, differences between clinical cohorts, and specific clinical values required to achieve optimum healthcare outcomes. Therefore, EBP and PM seem to present complementary approaches in contemporary, dynamically changing healthcare systems [1]. This was figuratively, and rather effectively, described by Spanakis et al. who explained that EBP leads to a situation where healthcare professionals see the forest (population averages), but fail to notice the trees (individual patients), while those utilising the PM strategy, “can’t see the forest for the trees” [2,3].

On the other hand, Personalised medicine (PM) and person-centered care (PCC) are two notions that in recent decades emerged independently to express ambitions to individualise the design of care and align it better to fit the situation of a specific patient. This again is in contrast to standardised guidelines and treatment prescriptions based on statistically average results from broad population studies. 

In line with all the aforementioned concepts, nursing staff (NS) play an important role in modern healthcare, as they advise, educate, and provide care for patients whose needs frequently require an individual approach (Personalised Nursing Care, PNC) [4]. A multitude of problems faced on a daily basis by nursing personnel include, among others, delirium which, being a serious condition, affects more and more patients, including those admitted to Intensive Care Units (ICUs). It is a serious acute neurocognitive condition frequently occurring in patients, yet it is often unrecognised [5]. There are many factors contributing to the development of delirium. These include sleep disturbance, pain, and most importantly pharmacotherapy used during treatment [1]. Nursing care for patients with delirium is extremely important not only for the patients themselves, but also for their families and the entire therapeutic team [1]. 

Delirium seems to be a common phenomenon observed in patients during the COVID-19 (Coronavirus Disease-2019) pandemic. SARS-CoV-2 (Severe Acute Respiratory Syndrome Coronavirus-2) was first identified in China in December 2019, and the epidemic of COVID-19 has now spread globally affecting many hospitals and patients [6]. Because of the type and spectrum of COVID-19 symptoms, and the still unknown nature of SARS-CoV-2, patients hospitalised and staying at ICU’s particularly require an individual and personalised approach. Furthermore, COVID-19 diagnosed in an ICU patient is not fully understood, while the provision of care is adversely affected by what is a particularly dangerous and highly infectious disease. Many nursing problems should therefore be considered on a case-by-case basis which requires an adjustment in the nature of nursing activities towards a patient with COVID-19 [7].

## 2. Non-Modifiable and Potentially Modifiable Risk Factors of Delirium in ICUs

Since all patients present individual characteristics, some are more prone than others to develop delirium. It has been shown that patients with delirium are more at risk of a prolonged hospital stay, functional and cognitive deterioration, as well as higher mortality rates and they are more likely to require institutional help. Delirium is an independent marker for 6- and 12-month mortality following hospitalisation [5]. Research findings show that mobility limitation, undernutrition, and inappropriate patient care provided by the therapeutic team are some of the most frequently reported risk factors of delirium. Furthermore, noise or lack of information concerning the activities that are being performed often result in increased fear, which may turn into delirium [8]. It has been shown that anxiety is one of the strongest contributing factors to the development of delirium in patients infected with the coronavirus [9]. The use of sedatives may also have a significant impact on the development of delirium [9]. According to the data reported in the literature, the older the patient, the higher the risk of delirium. Prolonged mechanical ventilation largely contributes to the development of delirium symptoms [10]. COVID-19 patients often require endotracheal intubation, performed in emergency setting when respiratory failure occurs. Hypoxia may then adversely affect their ability to understand explanations related to the activities performed as a result of which they may become disorientated, terrified, and even aggressive upon recovery from anaesthesia [6]. The isolation of patients and the ban on hospital visits during the COVID-19 pandemic increase the risk of anxiety and feeling of alienation, contributing to the development of delirium. The actions of the therapeutic team may increase or reduce the intensity of the symptoms contributing to the development of delirium. By maintaining contact with the patient and retaining self-control during nursing and therapeutic procedures, it is possible to calm the patient down and make them aware that they are treated in an individualised manner. Despite the ban on visits to patients in ICUs, it is important to maintain contact with the patients’ families via video calls or by passing on greetings to the patients. A customised approach to the patient has to be consistent with the therapy itself, e.g., if the patient suffers from delirium, sedatives should not be administered at first as they may lead to respiratory failure, and consequently to the need for endotracheal intubation, which is one of the elements contributing to the spread of the virus [11]. Governing bodies of the hospitals and wards intended only for patients with COVID-19 should pay particular attention to increasing the number of staff who take care of the patients. It should be emphasised that not only the above-mentioned issues, but also those related to the nurses themselves, such as burnout syndrome, weakness, and exhaustion may lead to serious consequences in nursing care. Despite the fact that it is known that personal protective equipment has to be used during work (full-body overalls, etc.), the stress associated with possible infection also produces a greater risk of developing burnout symptoms (Table 1) [11]. 

## 3. Tips for Nurses Managing Delirium

Nurses should not rush to diagnose a patient with delirium, but they should be aware that the patient may have it. Delirium may be a symptom of a life-threatening pathology. It is necessary to consider laboratory findings, imaging diagnostics, EEG (if epilepsy or nonconvulsive status epilepticus is suspected), and to remember that there are different forms of delirium: hypoactive—such patients are not diagnosed with delirium as they remain unproblematic, yet this form is more common in ICUs; hyperactive and mixed—delirium has frequently a multifactorial aetiology and every single cause should be checked. 

**Figure 1:**
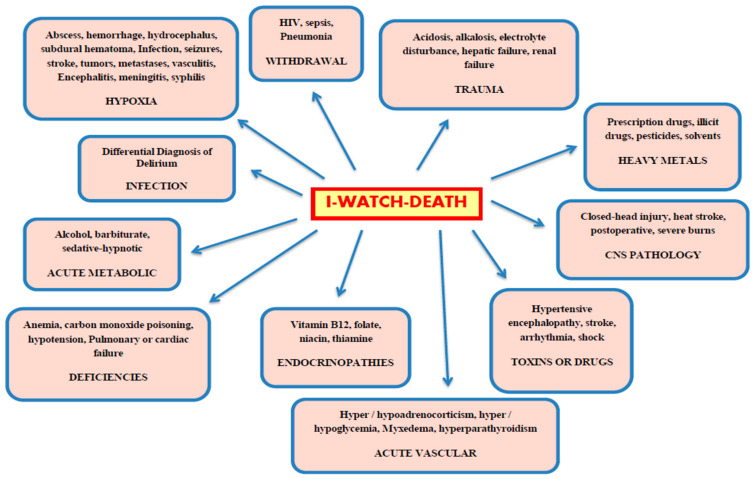
The I-WATCH-DEATH mnemonic [12].

The I-WATCH-DEATH mnemonic is widely used in clinical practice to help healthcare providers remember the common causes of delirium, and to help support bedside assessments. Figure 1 [12]. 

Modification of certain risk factors seems to reduce the incidence of delirium. The problem related to unknown surroundings may partly be solved with appropriate positioning of clocks, external windows, calendars with tagged dates, as well as verbal patient reorientation regarding events that are currently happening worldwide [13]. Cognitive stimulation may also prove helpful, especially if it involves regular visits, which are impossible during the pandemic due to the currently binding provisions of law. Another issue is the facilitation of physiological sleep. The volume of alarms should be decreased at night, the lights turned off, nursing interventions reduced, and ear stoppers used. A key role is attributed to pharmacological insomnia treatment, but errors should be avoided, i.e., insomnia should not be treated with drugs that cause delirium—benzodiazepines, diphenhydramine, and zolpidem. Administration of drugs late at night (2–5 a.m.) should be avoided, as it may lead to patients sleeping during the day. The preferred therapy includes quetiapine 25–50 mg orally (approximately 10–11 p.m.) and melatonin. Motor stimulation is also important—physiotherapy, early mobilisation, and the use of glasses and hearing aids in patients who normally would use them on a daily basis [13]. 

## 4. Tools Used in the Screening and Diagnostics of Delirium in Patients with COVID-19

**Figure 2:**
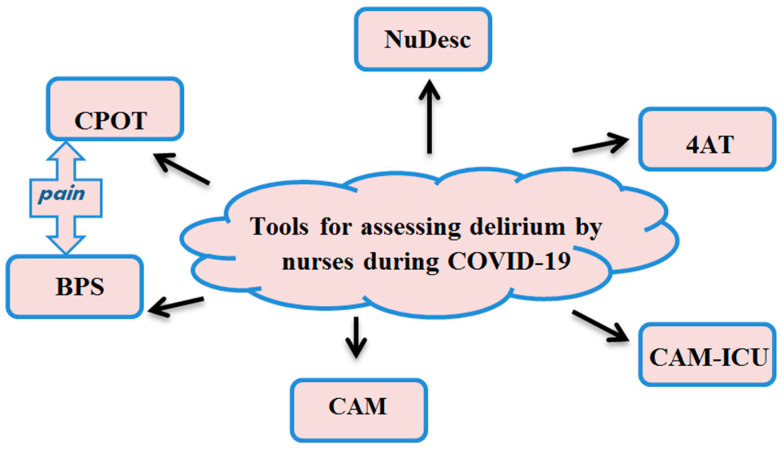
Scales, presented in the literature, recommended for use by nurses of patients with delirium [19].

Delirium screening is a very important part of the diagnostics process. The Richmond Agitation-Sedation Scale (RASS) should be the first scale to be used to assess the depth of sedation [14]. One of the best-known tools in delirium assessment is the Confusion Assessment Method (CAM) scale, which qualitatively evaluates disturbances in the state of consciousness [14]. Due to the severity of COVID-19, most patients are admitted to an ICU. The CAM tool was modified for the needs of ICU patients (Confusion Assessment Method for the ICU, CAM-ICU). The name suggests that the tool is used in an ICU setting to evaluate delirium in this population. Patients undergoing these tests should be able to understand the instructions presented by the staff. The CAM and CAM-ICU scales are more frequently employed by physicians [15]. Nurses, on the other hand, use a variety of other scales which can be successfully applied to identify the symptoms of delirium. One of these is the Nursing-Delirium Screening Scale (NuDesc) [16]. It should be underscored that a well-established workplan and carefully developed schedule of specific, personalised nursing activities may facilitate earlier recognition of delirium symptoms. Of prime importance is evaluation of the patient within 72 h from the onset of delirium symptoms. By ensuring appropriate selection of nursing processes based on evidence-based nursing it is possible to deliver adequate care at every stage of the patient’s hospital stay [17]. The 4 A’s test (4AT) is designed to be used by any health professional during first contact with a patient, and at any other time when delirium is suspected. 4AT has been translated into many languages [12]. It takes only two minutes to complete this test which may be used successfully with conscious patients. Patients with COVID-19 frequently suffer from respiratory failure, therefore it is important to measure oxygen saturation and to use the Glasgow Coma Scale (GCS) score to assess if the patient is capable of providing answers to the questions of the 4AT test [18]. The Delirium Observation Scale (DOS), a 13-item tool, which is fast to administer, captures the early symptoms of delirium that can be observed over the course of care, and does not necessarily require specialised training in geriatric care [19]. The PRE-DELIRIC (PREdiction of DELIRium in ICU patients) 10-item tool uses routinely available data collected within the first 24 h of admission to an ICU [20]. It assesses the risk of delirium developing in patients in critical care [20]. Furthermore, pain assessment is extremely important for identification of delirium in ICU patients, since the feeling of pain has a negative impact on the patient’s behaviour, and the intensity of pain is directly proportional to the onset of delirium [21]. Pain is also closely related to stress. According to the data reported in the related literature, the better the pain therapy, the better the outcome which may be expected in ICU patients [21]. According to current reports, appropriate scales, such as the Behavioural Pain Scale (BPS) [14,22] and Critical Care Pain Observation Tool (CPOT) [14,23], are the most appropriate for critically-ill patients. These scales are the most reliable tools in pain assessment in both non-invasively ventilated (NIV) and intubated patients. Moreover, they may be successfully used by ICU nurses. Early intervention to relieve the pain can minimise the risk of delirium [21]. Non-invasive ventilation (NIV) is commonly applied to prevent endotracheal intubation in patients with mild to moderate acute respiratory distress syndrome (ARDS) [24]. The RASS scale is worth mentioning as it is helpful in planning other nursing activities [25]. It assesses the consciousness of ICU patients, which is very important in patients with hypoxia, such as those suffering from respiratory failure in the course of COVID-19 (Figure 2) [19]. The aforementioned tools are recommended for use by advanced practice nurses (APN) who have expert knowledge and capabilities required to make decisions, and their skills and clinical competences related to advanced nursing are consistent with the highest standards.ór z uwagami recenzenta.

## 5. COVID-19 vs. Delirium in an ICU—What We Know and What We Do

**Figure 3:**
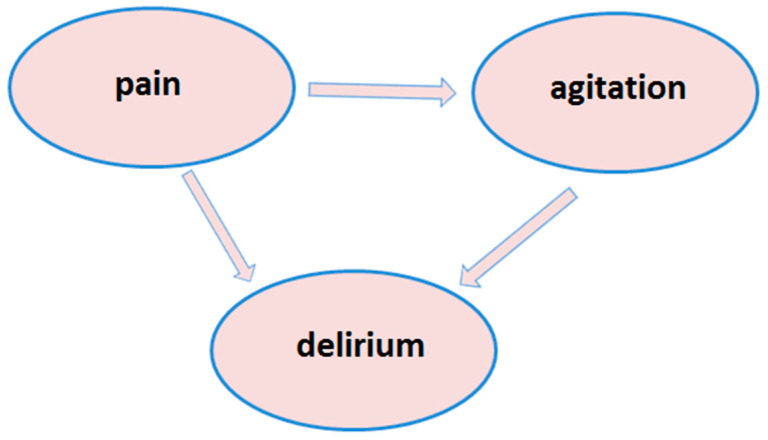
The Pain, Agitation, and Delirium (PAD) bundle [11].

The data available in the literature do not present a unified algorithm for treating patients with delirium during the COVID-19 pandemic. Researchers have shown that delirium may be treated in different ways by applying pharmacotherapy based for instance on benzodiazepines or opioids, or by using non-pharmacological interventions, depending on the condition of the patients and their reaction to the pharmacotherapy [26]. The Society of Critical Care Medicine (SCCM) named three factors which closely interact in adult ICU patients. These are pain, agitation, and delirium (PAD) (Figure 3) [11,27]. Awareness of the correlation between these three factors makes it possible to prevent each of them in order to minimise the risk of developing delirium.

Once these three elements are observed, the so-called ABCDEF bundle, consisting of six elements, should be immediately introduced. The ABCDEF bundle is an acronym for:

– **A**ssess, prevent and manage pain;
– **B**oth spontaneous awakening trials and spontaneous breathing trials;
– **C**hoice of sedation and analgesia;
– **D**elirium assessment, prevention and management;
– **E**arly mobility and exercise;
– **F**amily communication and involvement [28,29].

Research carried out by Balas et al. proved that the use of the ABCDEF bundle significantly reduces the probability of developing delirium. Furthermore, the use of this package has an impact on the onset of physical activity, from the moment of getting out of bed to independent functioning in everyday life [28].

Other methods which may be helpful for ICU patients suffering from COVID-19 is the spontaneous breathing trial (SBT) and spontaneous awakening trial (SAT) (Table 1; Supplementary Materials Figure S1) (Table 2) [30]. Both tests may be carried out in patients with well controlled pain. In such cases, attempts to discontinue sedatives and narcotics, as well as mechanical ventilation may prove successful. If both requirements are met, the patient may be allowed to “independently” wake up and breathe without the special equipment (Figure S2) [30]. 

**Figure 4:**
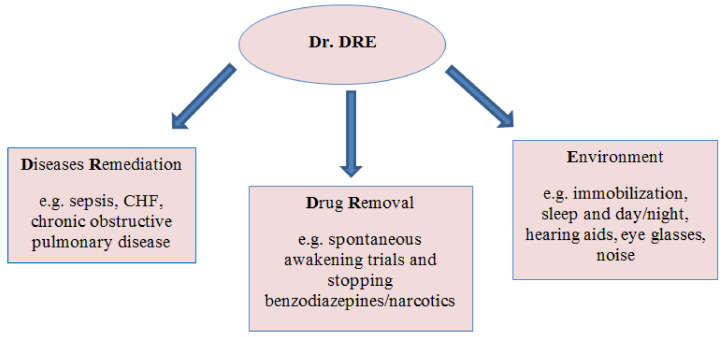
Simple mnemonic called “Dr. DRE” [30].

The agreed management algorithm gives the therapeutic team providing care to delirium patients a feeling of confidence in case a patient suddenly develops delirium. It is important to use checklists and discuss the risk factors observed by the team [27]. The Center for Health Services Research published a short algorithm which may be helpful for the entire team in determining the cause of delirium. Dr. DRE is an acronym for the three words to help the team (as presented in Figure 4 and Table 3) [30]. 

## 6. “Cooperative Sedation” and the Role of the Nurse

Current trends in ICU care, however, have shifted towards sedation strategies that provide the minimally effective amount of sedation to improve patient autonomy and preserve both the neurological exam and neurocognitive function [31,32]. Regardless of the etiology of neurocognitive deficits, strategies that target a lighter, more “cooperative” depth of sedation would allow the primary team to identify changes in mental status and cognition in the early phase of critical illness, when the condition may still be reversible. Sedation scales play a very important role in assessment and communication regarding sedation therapies available in ICU. There are many validated scales to assess the level of consciousness and agitation in patients requiring sedation. The Richmond Agitation-Sedation Scale (RASS) seems to be the most commonly used [14,33], while other available scales include the following: Riker Sedation-Agitation Scale (SAS) [34], Motor Activity Assessment Scale [35], Adaptation to the Intensive Care Environment (ATICE) [36] and Nursing Instrument for the Communication of Sedation (NICS) [37]. Despite the differences between these, the main aim of using sedation scales is to assess goal-directed sedation, personalised for each patient. One of the other objective tools for sedation assessment in critically-ill patients, which was also studied and has gained the most attention, is the bispectral index cerebral function monitor, which uses data based on raw electroencephalographic records (EEG) to quantitatively assess the patient’s agitation level. The bispectral index enables continuous monitoring of sedation with the additional benefit of attributing ordinal scores to relative EEG activity, which eliminates the subjective nature of sedation scales. This technology was used to assess the depth of anaesthesia during surgeries. The above-mentioned study proved that sedation reduced the number of episodes of intraoperative awareness [38]. 

Nursing support of sedation strategies is of key importance because it is the nurses who execute the sedation plan. Research findings indicate that most ICU nurses consider sedation necessary whenever the patient is intubated [39]. The impact of both personnel and pressure on other nursing activities was identified in one third of nurses. Being at the frontline of care, nurses belong to the group of attentive and compassionate healthcare practitioners, so the reason for light sedation and the likely need for the greater effort of nurses must be appropriately explained once the sedation plan is developed. However, light or “cooperative” sedation requires increased alertness, increased consciousness, and intellectual capabilities, which favour patient autonomy. Despite the potential need to use a means of physical coercion in some conditions, reduced sedation may allow patients to increase their involvement in the decision-making process regarding their own treatment [32]. 

## 7. ”Understanding Old Age” or Who Is Most at Risk?

An enormous threat to healthcare systems worldwide, COVID-19, affecting patients’ respiratory tract, puts the health of older adults particularly at risk. On the other hand, delirium is a well-recognised complication of respiratory illnesses in older patients, as well. Early studies suggest that 20–30% of COVID-19 patients will present with or will develop delirium or mental status changes during the course of their hospitalisation, with the rates of 60–70% in the case of severe illness at all ages [40,41]. Currently, assessment for COVID-19 does not routinely take into account delirium or mental status changes in older adults. Although the World Health Organization’s guidelines on suspected COVID-19 cases caution about “atypical symptoms” in the elderly and refer to possible “altered mental status”, they do not mention delirium explicitly [42]. Due to the fact that change in mental status is not routinely taken into account, it will be difficult, yet very important, to monitor its incidence as a major symptom, and identify its frequency particularly in the elderly. Delirium is considered to be a “barometer” or a “signature” of a serious condition in the elderly. Older patients with COVID-19 frequently do not present with the typical response reflected by fever and many of them do not show signs of breathlessness, even in the case of hypoxia [43]. 

O’Hanlon et al. in their article aptly pointed out the critical aspects of managing older individuals who constitute a majority of patients at ICUs [41]. Older people are most susceptible to severe COVID-19 infections and mortality. Current diagnostic guidelines do not routinely include delirium, which may result in underestimation of COVID-19 infections. Additionally, a failure to promptly detect COVID-19 may lead to outbreaks, e.g., among the elderly living in residential care facilities [7,42,43]. Older people are most vulnerable to severe COVID-19 infections and mortality. Current diagnostic guidance does not routinely include delirium, which may lead to under-detection of COVID-19. The care home population is particularly at risk, as failure to promptly detect COVID-19 may lead to local outbreaks. Non-pharmacological approaches to management of delirium may be more difficult to implement but remain the priority [41,42].

Non-pharmacological approaches to management of delirium may be more difficult to implement but remain the priority [41]. Researchers worldwide have taken steps which effectively resulted in excellent tools for combatting COVID-19 at every line of defence. Hospital Elder Life Program, recently combined with American Geriatrics Society CoCare Program (AGS CoCare: Help), has developed a Covid-19 Toolkit specifically designed to provide comprehensive resources for healthcare professionals to enable customized assessment and care of patients, and to ensure strategies for planning of treatments at acute, post-acute and long term stages and during palliative care (see https://help.agscocare.org/productAbstract/H00107). As a result, we can design personalised strategies for managing patients in every area, including ICUs. 

## 8. Personal Protective Measures versus Management of Patients with Delirium

It is highly probable that patients with delirium will not be able to follow rules of personal hygiene, which is of critical importance since the virus is highly infectious because large quantities of viral particles are discharged in body fluids [44]. According to researchers this is linked with the fact that the virus is protected by its robust outer shell against the activity of anti-microbial enzymes contained in the saliva and mucus [45]. Further, because SARS-CoV-2 has evolutionary links to burrowing animals that have association with buried faeces which could remain active in the environment for a long time, extra care is need when it comes to the cleaning of and caring for delirious patients [46]. 

## 9. Family, the Last but Not Least Important Element of the ABCDEF Bundle and the Personalised Approach to the Patient

Involvement of the family in patient care is a very important element of the ABCDEF bundle. During the COVID-19 pandemic, the presence of family members in hospitals is limited or even banned. In such cases the hospital staff should do their best to enable families to stay in touch with the patient. The cooperation of hospital staff with patients’ families, and an individualised patient approach allow for establishing contact with patients and mobilising them during this difficult period of recovery. The family should be educated and informed about the condition of the patient in such a way that they are not afraid during a telephone or video conference with the patient [47]. Personalised healthcare in nursing is an intangible asset, including explicit attributes (interprofessional collaboration and individualised care approach) and implicit attributes (managing personal vulnerabilities: molecular-based health information and self-health-seeking behaviours) [4]. It is necessary to consider the use of advanced technologies, computers, notebooks, and software at ICUs to enable communication between patients and their families. However, the healthcare personnel must also ensure that the presence of IT equipment does not contribute to the spread of SARS-CoV-2 to healthcare staff and to others outside the ICU. Supportive care should also be mentioned here, since this is a special type of care provided to patients and their families faced with a serious disease. It constitutes an additional layer of support for the care team and is provided along with the regular treatment. It has been shown that supportive care is indeed helpful, and the patients experience fewer symptoms, and their condition improves more rapidly. Supportive care of high quality involves basic, daily management which is required by any critically ill patient in order to prevent or overcome frequent problems. It is essential for every ICU patient. Minor interventions applied to thousands of patients may have a substantial overall impact.

## 10. Conclusions

In accordance with the assumptions of personalised healthcare, ICU nurses should have the knowledge and competences enabling them to understand, synthesise and utilise scientific advances, and to adjust their practice to the current and continuously changing global situation. Undergraduate and postgraduate nursing education, support in making clinical decisions, and changes in healthcare systems are all necessary to provide patients with individualised care where nurses are of key importance. Moreover, the job of a nurse involves work within an inter-professional team. 

It is necessary to “mobilise the resources” and urge international research teams to continue efforts. Global, multinational, multicentre research projects carried out by interdisciplinary teams will make it possible for us to “keep ahead of the enemy”. A fitting comment was offered by Tan et al.

Emergency grants would accelerate device production in view of the ongoing pandemic, similar to the additional publication of COVID-19 related research in medical literature. An extraordinary time in human history calls for special measures to match the needs of a shifting and transforming battleground. As various industries turn their efforts to addressing the needs of health care, those on the ground should be equipped to contribute their first-hand expertise by all means possible [48,49].

## Figures and Tables

**Table 1:** Risk factors for the development of delirium in an Intensive Care Unit (ICU) [11].

Non-Modifiable	Potentially Modifiable
Dementia and cognitive disturbances	Immobilisation (catheters, probes, tubes, intravenous infusions, restraint straps)
History of delirium	Medication (sedatives, narcotics, anticholinergics, corticosteroids, complex pharmacotherapy, sudden discontinuation of certain drugs and benzodiazepines)
History of stroke and neurological disease	Acute neurological diseases (acute stroke, especially within the right parietal region, intracranial bleeding, meningitis, encephalitis)
Falls and gait disturbances	Comorbidities (infections, acute diseases, anaemia, dehydration, undernutrition, fracture, and trauma)
Advanced age	Vision and hearing impairment
Complex multimorbidity	Metabolic disturbances (hypoglycaemia, hyperglycaemia, hyponatremia, hypernatremia, etc.)
Male gender	Surgeries
Chronic renal or liver disease	Environmental (e.g., admission to the ICU)
	Pain
	Stress, long-lasting insomnia

**Table 2:** The “Wake Up and Breathe” protocol [29].

**SAT Safety Screen**	**SBT Safety Screen**
No active seizures No alcohol withdrawal No agitation No paralytics No myocardial ischemia Normal intracranial pressure	No agitation Oxygen saturation ≥ 88% FiO_2_ ≤ 50% PEEP ≤ 7.5 cm H_2_O No myocardial ischemia No vasopressor use Inspiratory efforts
**SAT Failure**	**SBT Failure**
Anxiety, agitation, pain Respiratory rate > 35/min Oxygen saturation < 88% Respiratory distress Acute cardiac arrhythmia	Respiratory rate > 35/min Respiratory rate < 8/min Oxygen saturation < 88% Respiratory distress Mental status change Acute cardiac arrhythmia

**Table 3:** Explanation to Figure 4.

Remove deliriogenic medications—substitute medications such as benzodiazepines, anticholinergic medications (H2 blockers), steroids, etc. Non-pharmacological interventions Analgesia—adequate pain control may decrease delirium. Consider intermittent morphine administration, if feasible. Atypical or typical antipsychotics—one may consider 1–2 mg haloperidol as a starting dose in the elderly. Maximum dose is usually 20 mg/day of haloperidol. Monitor ECG (electrocardiogram). Spontaneous Awakening Trial (SAT)—stop sedation or decrease infusion by ½, especially benzodiazepines, till RASS (Richmond Agitation-Sedation Scale)—to −2, as tolerated. Spontaneous Breathing Trial (SBT)—CPAP/PS (Continuous Positive Airway Pressure) trial if on <50% and ≤PEEP. S&A—sedative and analgesic drugs—commonly benzodiazepines, Propofol, fentanyl or morphine.	Non-pharmacological interventions. *Orientations* Provide visual and hearing aids. Encourage communication and orientation to the day/time/location by nurses and family. Have familiar objects from the patients’ home in the room. Attempt consistency in nursing staff. Allow television during the day with daily news. Non-verbal music. *Environment* Sleep hygiene: lights off at night, on during the day. Consider sleep aids (zolpidem, mirtazapine). Control excess noise (staff, equipment, visitors) at night. Ambulate or mobilize patients. *Clinical parameters* Maintain systolic blood pressure >90 mmHg. Maintain saturations >90%. Treat underlying metabolic derangements and infections. Discontinue any unnecessary and potentially deliriogenic medications.

## Supplementary Materials

The following are available online at https://www.mdpi.com/2227-9032/8/4/578/s1. Figure S1: Diagram of the procedure in the “Wake Up and Breathe” Protocol, Figure S2: Delirium protocol—workflow.
