# Supplementary material for: Nursing Interventions to Prevent Delirium in Critically Ill Patients in the Intensive Care Unit during the COVID19 Pandemic—Narrative Overview
Source: Healthcare (Basel). 2020 Dec 21;8(4):578. doi: 10.3390/healthcare8040578 (PMC7766119; doi:10.3390/healthcare8040578)
Supplement: Supplementary file 1 [file healthcare-08-00578-s001.pdf]

healthcare

MDPI

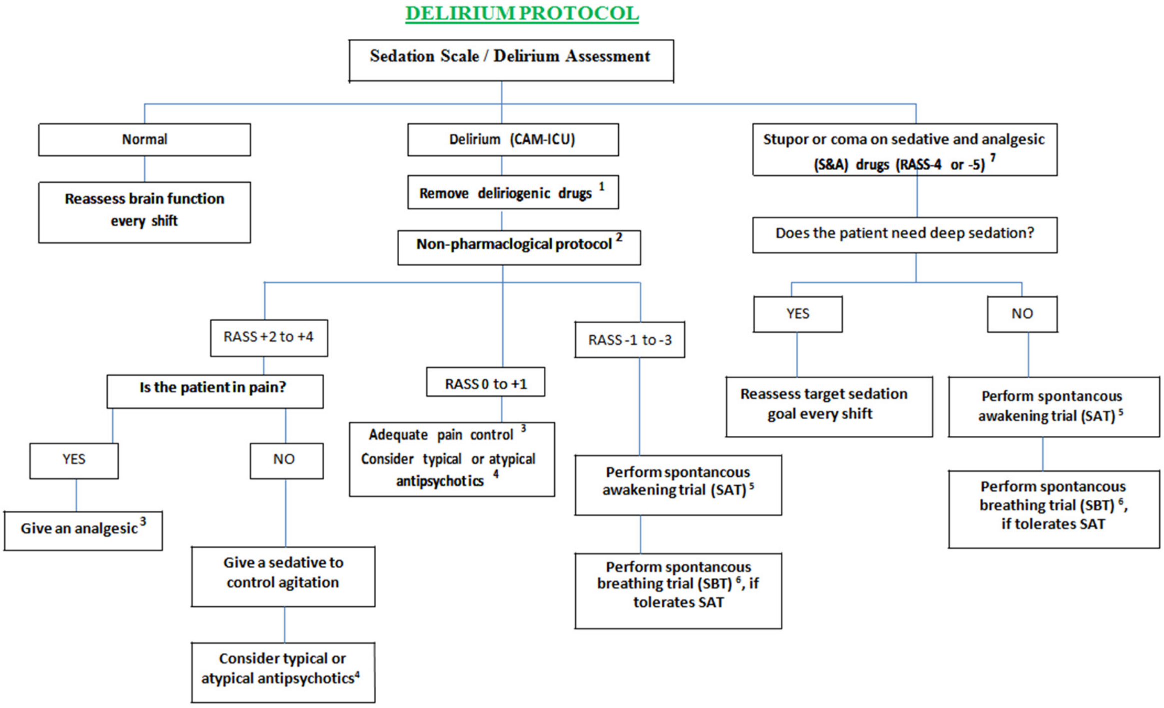

Figure 1. Delirium protocol – workflow.

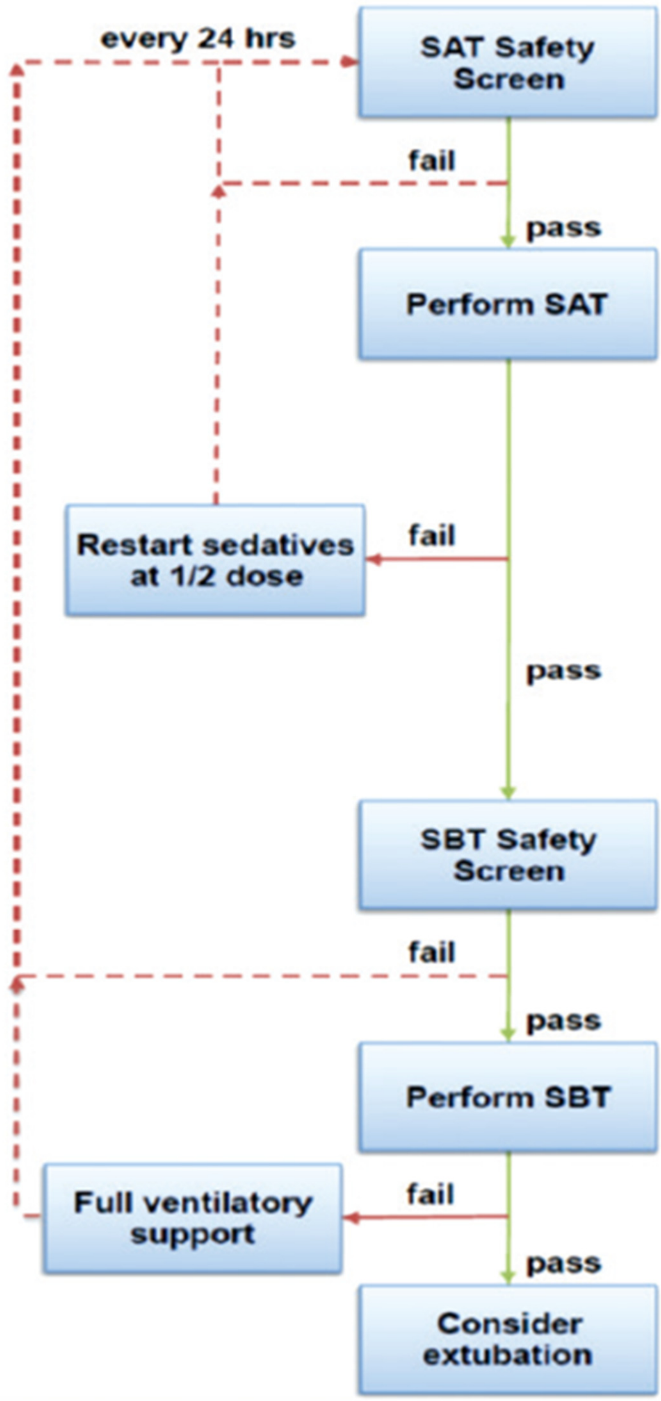

Figure 2. Diagram of the procedure in the 'Wake Up and Breathe' Protocol.
